# Radial Artery Access for Percutaneous Cardiovascular Interventions: Contemporary Insights and Novel Approaches

**DOI:** 10.3390/jcm8101727

**Published:** 2019-10-18

**Authors:** Renato Francesco Maria Scalise, Armando Mariano Salito, Alberto Polimeni, Victoria Garcia-Ruiz, Vittorio Virga, Pierpaolo Frigione, Giuseppe Andò, Carlo Tumscitz, Francesco Costa

**Affiliations:** 1 Department of Clinical and Experimental Medicine, Policlinic “G. Martino”, University of Messina, 98100 Messina, Italy; rfm.scalise@gmail.com (R.F.M.S.); armandosalito@gmail.com (A.M.S.); vittorio.virga@yahoo.it (V.V.); pfrigione@homail.com (P.F.); gando@unime.it (G.A.); 2 Division of Cardiology, Department of Medical and Surgical Sciences, Magna Graecia University, 88100 Catanzaro, Italy; polimeni@unicz.it; 3 UGC del Corazón, Servicio de Cardiología, Hospital Clínico Universitario Virgen de la Victoria, 29010 Málaga, Spain; mavigaru@gmail.com; 4 Department of Cardiology, Azienda Ospedaliero Universitaria di Ferrara, Via Aldo Moro, 8, Cona, 30010 Ferrara, Italy; Tumscitz@gmail.com

**Keywords:** radial, ulnar, distal radial, snuffbox, percutaneous coronary intervention, aortic valvuloplasty

## Abstract

Since its introduction, the transradial access for percutaneous cardiovascular procedures has been associated with several advantages as compared to transfemoral approach, and has become the default for coronary angiography and intervention. In the last 30 years, a robust amount of evidence on the transradial approach has been mounted, promoting its diffusion worldwide. This article provides a comprehensive review of radial artery access for percutaneous cardiovascular interventions, including the evidence from clinical trials of transradial vs. transfemoral approach, technical considerations, access-site complications and limitations, alternative forearm accesses (e.g., ulnar and distal radial artery), and ultimately the use of the radial approach for structural interventions.

## 1. Introduction

The radial artery has progressively become during the last decade the standard access site for percutaneous coronary diagnostic and interventional procedures. The first report of transradial access (TRA) for cardiovascular diagnostics dates to 1947, when Radner performed an aortography via radial artery employing a cut-down technique [1]. Afterwards, in 1989, Campeau published the first case series of 100 transradial coronary angiographies performed through the radial access, emphasizing the safety and the fast mobilization of patients with this approach [2]. Hereafter, in 1993, Kiemeneij described three cases of transradial coronary stenting [3]. On the basis of this pioneering work, TRA gradually diffused supported by a growing body of evidence. As compared to the transfemoral approach (TFA), the radial artery has a more superficial location that allows an easier localization and compression resulting in a lower rate of hemorrhagic complications. Furthermore, the absence of damageable structures reduces the risk of complications due to the puncture itself, while the vascular reserve provided by ulnar artery limits distal ischemic injury of the hand. In addition, the radial access improves patient comfort and accelerates mobilization. On the contrary, radial interventions may result tricky in case of tortuous anatomies of the subclavian axis and results in a more difficult coronary cannulation and catheter manipulation. This results in a significant learning curve for operators that trained with femoral approach as a standard access site. While radial access provides undoubted advantages in most of coronary interventional cases, femoral access remains the preferred route for complex high-risk coronary interventions or when bulky devices are necessary (e.g., mechanical circulatory support, structural procedures etc.). The purpose of this review is to provide a comprehensive overview of the evidence, technical considerations, and future perspectives of the radial artery and other forearm arterial approaches for cardiovascular interventions.

## 2. Evidence Supporting Transradial Artery Access

A solid body of evidence have mounted in the last 10 years regarding the advantages provided by the radial approach for coronary interventions (Table 1). The Mortality benefit Of Reduced Transfusion after percutaneous coronary intervention via the Arm or Leg (MORTAL) registry [4], a large retrospective study including 30,900 patients treated with percutaneous coronary intervention (PCI), showed for the first time a reduced need for blood transfusion and 1-year mortality with TRA as compared to TFA. These preliminary findings were confirmed by other observational studies. The lack of randomized evidence confronting radial vs. femoral approach was filled by the RadIal Vs. femorAL access for coronary intervention (RIVAL) trial [5], the first randomized comparison between TRA and TFA for PCI in patients with acute coronary syndromes. The RIVAL trial enrolled 7021 patients, which were randomly assigned to TRA (*N* = 3507) or TFA (*N* = 3514) at the time of vascular access. The primary endpoint, a composite of death, myocardial infarction, stroke, or non-coronary artery bypass graft (CABG)-related major bleeding at 30 days, was similar in both groups (3.7% in TRA vs. 4.0% in TFA; HR 0.92, 95% CI 0.72–1.17; *P* = 0.50). No difference for ischemic endpoints was observed between the two treatment arms, neither a difference in terms of the primary safety endpoint of non-CABG major bleeding. Post-hoc analysis including an alternative definition of bleeding showed a significant reduction of major bleeding with TRA. Major vascular complications were more common in the TFA group (1.4% vs. 3.7%; *P* < 0.0001), whereas the need for access-site crossover (i.e., transition between the randomly assigned access to an alternative access) was more common in the TRA group (7.6% vs. 2.0%; *P* < 0.0001). The subgroups analysis of the primary endpoint revealed a significant interaction favoring TRA over TFA in highest tertile volume radial centers (HR: 0.49, 95% CI: 0.28–0.87; *P* = 0.015) and in patients presenting with ST-segment elevated myocardial infarction (STEMI) (HR: 0,60, 95% CI: 0.38–0.94; *P* = 0.026). In particular, in patients with STEMI approached via TRA a lower rate of the composite of death, MI and stroke (P int = 0.011), and death for all causes (P int = 0.001) was also observed, in line with the hypothesis that a more intensive antithrombotic regimen implemented in STEMI patients could unveil the benefit of the radial access.

The overall neutral findings of the RIVAL trial contrasted with the previous evidence from observational studies. A possible explanation for this inconsistency could be found in a deeper evaluation of this study. A subsequent analysis of the RIVAL trial showed that all the access-site major bleeding reported in the radial arm were due to intra-aortic balloon pump use and radial-to-femoral crossover, which were assigned to the radial cohort in keeping with the intention-to-treat analysis. After relocating these events in the femoral group in the as-treated analysis, a significant relationship between femoral approach and access-site major bleeding was found also for the study safety endpoint (0 vs. 18 events) [5]. Finally, the RIVAL trial may have been underpowered to observe differences in non–CABG-related major bleeding, hence reducing its ability to find superiority for the radial approach on the primary endpoint [5].

In line with the STEMI subgroup analysis of the RIVAL trial, the Radial versus Femoral Randomized Investigation in ST-Elevation Acute Coronary Syndrome (RIFLE-STEACS) trial, tested the superiority of TRA vs. TFA in patients presenting with STEMI [6]. This study enrolled 1001 patients undergoing primary or rescue PCI. The primary endpoint of the study was a composite of death, myocardial infarction, target lesion revascularization, stroke or non–CABG-related major bleeding defined by Bleeding Academic Research Consortium (BARC) at 30-days. TRA was ultimately associated with a significant reduction of the primary endpoint (13.6% in TRA vs. 21% in TFA; *P* = 0.003), reduced non–CABG-related major bleeding (7.8% in TRA vs. 12.2% in TFA; *P* = 0.026) and overall mortality (5.2% in TRA vs. 9.2% in TFA; *P* = 0.02). These impressive results should be closely analyzed to understand major differences with the RIVAL study. All operators in the RIFLE-STEACS were experienced and highly skilled performing more than 200 PCI per year with the radial approach. Furthermore, the mortality and the bleeding event-rate observed in the RIFLE-STEACS population was higher compared to previous studies, likely owing to the inclusion of a higher risk population with extensive use of GPIIb/IIIa inhibitors. Moreover, a subsequent analysis using the more stringent Thrombosis in Myocardial Infarction (TIMI) bleeding definition revealed no difference in access-site bleeding among the two treatment arms [6].

Subsequently, the STEMI-RADIAL trial, a multicenter randomized study, compared TRA and TFA in patients undergoing primary PCI performed by high-volume operators experienced in both access sites. The study enrolled 707 patients with STEMI who were randomized to TRA (*N* = 348) or TFA (*N* = 359) before the primary PCI [7]. The primary endpoint, a composite of major bleeding and vascular access-site complications at 30 days, was significantly lower in the radial cohort (1.4% vs. 7.2%; *P* < 0.0001). Likewise, the net adverse clinical events (NACE) rate, a composite of death, myocardial infarction, stroke, major bleeding and vascular complications was lower in the radial cohort (4.6% vs. 11%; *P* = 0.0028). The mortality at 30 days was similar in both group (2.3% in the TRA group vs. 3.1% in TFA group; *P* = 0.64).

A paramount contribution in the field was given by the Minimizing Adverse Hemorrhagic Events by TRansradial Access Site and Systemic Implementation of angioX (MATRIX) program [8,9]. This large multicenter study was designed to assess if TRA or bivalirudin, compared with TFA or unfractionated heparin, decrease the 30-day incidence of ischemic or and/or bleeding events in patients with acute coronary syndrome undergoing early invasive management. The MATRIX access [10], the first trial from this program, was a multicenter superiority trial comparing TRA versus TFA. The study population was composed of 8404 patients with acute coronary syndrome, with or without ST-segment elevation myocardial infarction, undergoing coronary angiography and PCI which were randomized to TRA (*N* = 4197) or TFA (*N* = 4207). The two co-primary endpoints were Major Adverse Cardiovascular Events (MACE) at 30 days and NACE at 30 days using the BARC major bleeding definition. Results showed a similar MACE rate in both groups (8.8% in TRA group vs. 10.3% in TFA group; RR: 0.85, 95% CI: 0.74–0.99; *P* = 0.0307, non-significant α of 0.025) whereas NACE rate was lower in patients of TRA group (9.8% vs. 11.7%, RR: 0.83, 95% CI: 0.73–0.96; *P* = 0.092). The benefit in radial access was mainly due to a significant reduction of non-CABG-related major bleeding (1.6% in TRA group vs. 2.3%, RR: 0.67, 95% CI: 0.49–0.92; *P* = 0.013) and all-cause mortality (1.6% in TRA group vs. 2.2% in TFA group, RR: 0.72, 95% CI: 0.53–0.99; *P* = 0.045). Results remained consistent also at a longer-term follow-up of one year [11].

A meta-analysis of the these four multicenter randomized trials (RIVAL, RIFLE-STEACS, STEMI-RADIAL, and MATRIX access) including a total of 17,133 patients (8552 in TRA group and 8581 in TFA group) demonstrated that TRA reduced mortality (RR: 0.73, 95% CI: 0.59–0.90; *P* = 0.003), major adverse cardiovascular events (RR: 0.86, 95% CI: 0.75–0.98; *P*= 0.025) and major bleeding (RR: 0.57, 95% CI: 0.37–0.88, *P* = 0.011) with no effect on myocardial infarction or stroke. Yet, a minimal procedural time delay (standardized mean difference of 0.11 min; 95% CI: 0.04–0.18 min; *P* = 0.002) and a higher rate of access-site crossover (6.3% vs. 1.7% *P* = 0.001) was associated with TRA [12]. These results remained consistent in a second subanalysis including also observational studies [13].

Recently, the SAFARI-STEMI trial, presented at the American College of Cardiology in 2019 [14] compared TRA and TFA in 4884 patients undergoing primary PCI for STEMI. At interim analysis, due to lower than expected rate of the primary outcome, the study was terminated for futility. Finally, just 2292 patients were enrolled a randomized to TRA (*N* = 1136) or TFA (*N* = 1156). Results showed no difference in 30-day all-cause mortality (1.8% in TRA group vs. 1.6% in TFA group; *P* = 0.83), reinfarction (1.8% in TRA group vs. 1.6% in TFA group; *P* = 0.83), stroke (1.0% in TRA group vs. 0.4% in TFA group; *P* = 0.12) and a composite endpoint including death, reinfarction and stroke (4.0% in TRA group vs. 3.4% in TFA group; *P* = 0.45). Unexpectedly, the bleeding rate was comparable between the two groups independently from bleeding definition used (1.1% in TRA group vs. 1.3% in TFA group; *P* = 0.74).

## 3. Technical Factors and Access-Site Complications

**Figure 1:**
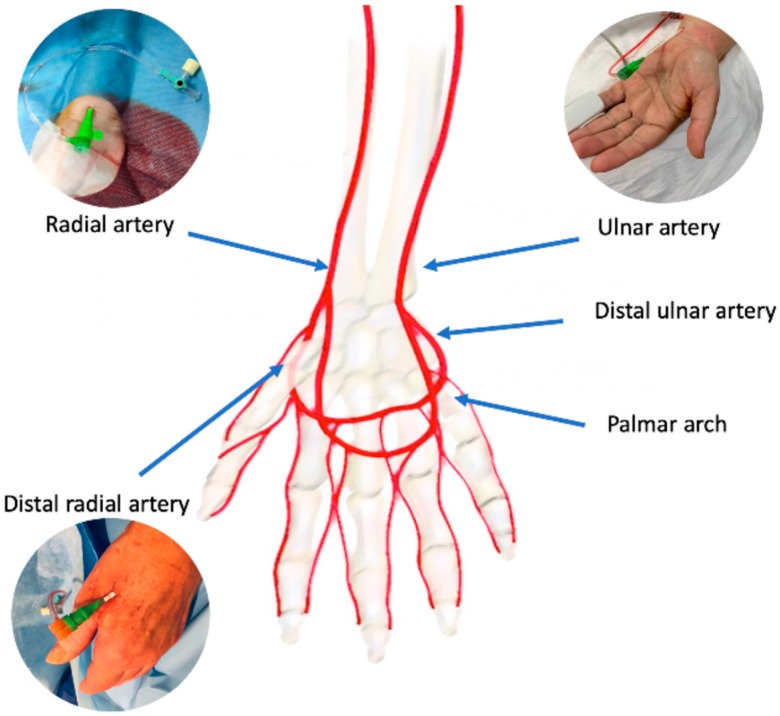
Arterial anatomy of the wrist/hand and potential vascular accesses.

The radial artery originates, together with the ulnar artery, from the brachial artery bifurcation at the proximal border of the antecubital fossa (Figure 1).

The radial artery can be divided in three segments: the proximal segment laying in the forearm, the middle segment running in the back of the wrist, and the distal segment transitioning from the wrist to the dorsal side in the hand through the anatomical snuffbox. An intricate anastomotic network formed by the deep palmar arch, deriving mainly from the radial artery, and the superficial palmar arch deriving mainly by the ulnar artery provides the hand a dual blood supply. As observed in post-mortem studies [15] this anastomotic circle can present highly variable patterns. Sometimes, a superficial palmar branch can stem from the radial artery in the forearm and it can converge in the distal ulnar artery segment, completing the superficial palmar arch; in the same way, a deep palmar branch can stem from distal ulnar artery and it can converge in the distal radial artery completing the deep palmar arch. Theoretically, the patency of palmar connection between radial and ulnar artery is essential to prevent hand ischemic complications if the radial or the ulnar artery occludes. Several non-invasive tests have been proposed to evaluate the palmar arch circulation: the Allen test, the modified Allen test, and the Barbeau test. In the modified Allen test, the patient first has to lift the hand and clench the fist, then the operator compresses both radial and ulnar artery to block the flow and finally the patients opens the fingers, the operator removes ulnar artery compression and counts the time necessary to recolor the hand. The test is normal if blushing appears within 5 s, it is intermediate if blushing appears between 6 and 10 s, it is abnormal if blushing takes more than 10 s to appear. Similarly, the Barbeau test is performed in a similar manner as the modified Allen test but implements the evaluation of the plethysmographic waveform and pulse oximetry evaluation of the hand [16]. Recently, the laser Doppler scan, a novel promising non-invasive diagnostic technique, has been described [17]. It uses a low-power laser beam and generates color maps of blood perfusion. This test allows quick and easy diagnosis of radial artery occlusion after catheterization in an operator-independent manner [18].

Nevertheless, the effectiveness of these non-invasive tests is debatable and did not appear essential to prevent hand ischemia.

The Predictive Value of Allen’s Test Result in Elective Patients Undergoing Coronary Catheterization Through Radial Approach (RADAR) study [19] evaluated prospectively the safety and feasibility of TRA approach in relation to the Allen test. The study enrolled 203 patients undergoing elective or urgent coronary catheterization which were divided based on Allen test results in three groups: normal (*N* = 83), intermediate (*N* = 60) and abnormal (*N* = 60). Every patient was subjected to serial assessments of thumb capillary lactate (the primary endpoint), thumb plethysmography, ulnar frame count, handgrip strength tests, and discomfort ratings. No statistically significant difference emerged among the 3 study groups for lactate (mean: 1.85 ± 0.93 mmol/L in normal group, 1.85 ± 0.66 mmol/L in intermediate group, and 1.97 ± 0.71 mmol/L in abnormal group; *P* = 0.59), handgrip strength test results, and discomfort ratings after the procedure or at other time points during the study. In patients with abnormal or intermediate results, plethysmography revealed ulnopalmar collateralization improvements while the ulnar frame count was reduced, indicating augmented ulnar flow after TRA.

The ACRA anatomy study (Assessment of Disability After Coronary Procedures Using Radial Access) [20] evaluated the clinical relevance of the palmar arch incompleteness and the correlation to commonly used patency tests. This study enrolled 234 patients undergoing TRA cardiac catheterization. Patients were valued angiographically to define the hand vascular anatomy and with modified Allen test and Barbeau test before TRA. Furthermore, a functional evaluation of the hand was assessed at baseline and 2-year follow-up by the QuickDASH, a shortened version of the 30-item DASH (Disabilities of the Arm, Shoulder and Hand) questionnaire. Results showed that incompleteness of the superficial palmar arch was present in 46% of patients, meanwhile the deep palmar arch was complete in all patients. Modified Allen test and Barbeau test results were associated with incompleteness of the superficial palmar arch (*P* = 0.001 and *P* = 0.001). The modified Allen test sensitivity and sensibility for superficial palmar arch incompleteness were 33% and 86% respectively with a cut-off of >10 s and 59% and 60% respectively with a cut-off of >5 s. The Barbeau test sensitivity and specificity were 7% and 98% respectively for type D and a 21% and 93% respectively for types C and D combined. The upper-extremity dysfunction had no correlation with superficial palmar arch incompleteness (*P* = 0.77). These results suggested that while the incompleteness of the superficial palm arch is common, a complete deep palmar arch guarantees the blood supply to distal districts, and the preprocedural patency tests do no add benefit to prevent ischemic complications of the hand.

The ACRA-perfusion [21], a prespecified sub-study of the ACRA anatomy study, focused on how transradial approach may impair digital perfusion. Sequential laser Doppler perfusion imaging was used to evaluate digital perfusion in 100 patients undergoing elective transradial cardiac catheterization in comparison with contralateral hand. The primary outcome was thumb perfusion at baseline, during radial access, at TR band application and at discharge. The vascular anatomy was assessed with angiography and the upper-extremity function was measured by QuickDASH questionnaire at baseline and follow-up. Results showed a reduced tissue perfusion during radial access and during TR band application both in the homolateral thumb (−32% and −32%, respectively) than in contralateral thumb (−34% and −21%, respectively). No perfusion difference emerged during TRA between the homolateral and the contralateral thumb (arbitrary flux units: 217; IQR, 112–364 vs. 209; IQR, 99–369; *P* = 0.59). There was no association between reduced thumb perfusion and superficial palmar arch incompleteness (complete superficial palmar arch 56% vs. incomplete superficial palmar arch 71%; *P* = 0.13). Digital perfusion at discharge remained below baseline levels but showed an improving trend (homolateral −11% and contralateral −14%). Reduced digital perfusion during TRA was not associated with hand dysfunction at 18 months.

The radial approach procedure begins with the administration of local anesthetic to limit the patient’s discomfort, usually a modest dose (1–2 mL) is used to not distort the normal anatomy. The radial artery cannulation is commonly obtained close to the styloid process with two different techniques: the Seldinger and the modified Seldinger technique [22]. The Seldinger technique employs a catheter-over-needle system which is used first to pass the anterior wall and subsequently, when blood fills the needle, the posterior wall; the needle is then removed and the catheter is slowly draw back until a pulsatile blood flow is obtained; finally a 0.021-inch guidewire is advanced, the catheter is removed, and the introducer over the wire is placed. The modified Seldinger technique employs a micro-puncture needle entering arterial lumen, as soon the blood flows through the needle a 0.018 inch or 0.021 inch guidewire is advanced into the vessel, the needle is removed, and an introducer over the wire is placed. A randomized evaluation of these two techniques was performed in 412 patients undergoing TRA catheterization [23]. 210 patients were approached with the Seldinger technique and 212 with the modified Seldinger technique. Results showed a statistically significant advantage for the Seldinger technique regarding time to gain radial artery access (mean time in seconds 78.3 ± 37.7 vs. 134.2 ± 87.5; *P* < 0.001) and total procedure time (mean time in minutes 17.1 ± 6.4 vs. 19.3 ± 7.1; *P* < 0.01), while no difference emerged in post-procedural hematoma (0.5% vs. 1.5%, *P* > 0.2), and radial artery occlusion at 24 h (8% vs. 7.9%; *P* > 0.5) or at 30 days (4.3% vs. 3.9%; *P* > 0.5). A meta-analysis comparing all available studies on the use of 5-Fr versus 6-Fr system in coronary procedures through the TRA showed a significant lower contrast medium administration (MD = −22.20 (−36.43 to −7.9), *P* < 0.01) and bleedings (OR = 0.58 (0.38–0.90), *P* = 0.02) of 5-Fr system, without compromising procedural success (OR = 0.95 (0.53–1.69), *P* = 0.86) or procedure length (OR = 0.55 (−2.58 to 3.69), *P* = 0.73), compared to the 6-Fr system [24].

Introducer sheaths of different sizes are available on the market and many operators usually prefer longer sheaths (23 cm) as compared to short sheaths (13 cm) due to a supposed spasm reduction and easier catheter manipulation. A randomized controlled trial investigated the impact of length and hydrophilic coating of the introducer sheath on radial artery spasm, radial artery occlusion, and local vascular complications in patients undergoing elective transradial coronary procedures [25]. This study enrolled 790 patients, which were randomly assigned to long (23-cm) or short (13-cm) and hydrophilic-coated or uncoated introducer sheaths. Results showed a reduction of radial artery spasm (19.0% vs. 39.9%, OR: 2.87; 95% CI: 2.07 to 3.97; *P* = 0.001) and patient reported discomfort (15.1% vs. 28.5%, OR: 2.27; 95% CI: 1.59 to 3.23; *P* = 0.001) in patients receiving a hydrophilic-coated sheath while no difference emerged between long and short sheaths.

The R-RADAR study [26] (The Rotterdam Radial Access Research Ultrasound-Based Radial Artery Evaluation for Diagnostic and Therapeutic Coronary Procedures) investigated structural changes of the radial artery wall after catheterization to define predictors of radial pulsation loss, occlusion, local pain, or functional impairment of the upper extremity. This prospective, single-center study enrolled 90 patients undergoing TRA coronary angiography or intervention, which were scanned with a high-resolution 40-MHz ultrasound (US) before cannulation, at 3 h and 30 days after procedure. All patients presented acute injuries, predominantly dissection and intramural hematoma, but these were not associated with loss of radial pulsation, occlusion, local pain, or functional impairment at 30 days. An intimal thickening and total wall thickness increase was observed at 3 h after puncture and persisted at 30 days, while a modest luminal inner caliber reduction was observed at the puncture site. Radial occlusion and pulsation loss rate at 30 days were 3.9% and 9.2% respectively. Smaller radial artery lumen at baseline was associated with an increased the risk of radial pulsation loss at 30 days (OR 1.23; *P* = 0.049). The number of radial puncture attempts was associated with 30-day pulsation loss (OR, 2.64; *P* = 0.027), artery occlusion (OR, 3.49; *P* = 0.022), and symptoms (OR 2.24; *P* = 0.05).

The utility of US for guiding TRA was evaluated in the RAUST study (Radial Artery access with Ultrasound Trial) [27]. This prospective multicenter trial enrolled 698 patients undergoing transradial cardiac catheterization that were randomized to needle insertion with palpation (*N* = 351) or real-time US guidance (*N* = 347). The primary endpoints were the first-pass success rate, the total number of attempts needed for access, and the time to access. US guidance was related to a number attempts reduction mean: 1.65 ± 1.2 vs. 3.05 ± 3.4; *P* < 0.0001; median: 1 (IQR: 1 to 2) vs. 2 (1 to 3); *P* < 0.0001 and to a first-pass success rate improvement (64.8% vs. 3.9%; *P* < 0.0001) with a consensual time to access reduction (mean: 88 ± 78 s vs. 108 ± 112 s; *P* = 0.006; median: 64 (IQR: 45 to 94) vs. 74 (IQR: 49 to 120); *P* = 0.01). Crossover from palpation to US guidance was necessary in 10 patients with subsequent successful sheath insertion rate of 80%. US guidance also reduced the difficult access rate procedures (2.4% vs. 18.6% for ≥5 attempts; *P* < 0.001; 3.7% vs. 6.8% for ≥5 min; *P* = 0.07).

The right-sided controls position in the catheterization laboratory predisposes to a right radial approach (RRA) that both operator and the patients generally prefer [28]. Despite this, the left arterial access (LRA) offers a direct entrance to the left subclavian artery with a better support for guides or catheters and ease left internal mammary artery cannulation. Furthermore, the LRA has been associated with a reduced procedural duration and a higher rate of procedural success mainly due to the low prevalence of left subclavian artery tortuosity. Controversial data emerged comparing right and LRA about radiation exposure duration.

The Transradial approach left vs. right and procedural times during percutaneous coronary procedures (TALENT) [29] study evaluated the safety and efficacy of LRA compared with RRA for coronary procedures focusing on fluoroscopy time for coronary angiography and PCI (primary endpoints). Patients age and operator experience were also analyzed. This dual-center study enrolled 1540 patients which were randomized to RRA (*N* = 770) or LRA (*N* = 770); 1467 patients underwent coronary angiography (diagnostic group: 732 with LRA approach and 735 with RRA) and 688 patients underwent percutaneous coronary procedures (PCI group: 344 each group). Fluoroscopy time (median: 149 s, IQR 95–270 s), as well as Dose Area Product (DAP) (median: 10.7 Gy cm^2^, IQR 6–20.5 Gy cm^2^), were significantly lower with LRA in the diagnostic group when compared with RRA (median: 168 s, IQR 110–277 s; *P* = 0.0025 and 12.1 Gy cm^2^, IQR 7–23.8 Gy cm^2^; *P* = 0.004, respectively). No significant differences emerged in the PCI group in fluoroscopy time (median: 614 s, IQR 367–1087 s for LRA vs. 695 s for RRA, IQR 415–1235 s; *P* = 0.087) and DAP (median: 53.7 Gy cm^2^, IQR 29–101 Gy cm^2^ for LRA vs. 63.1 Gy cm^2^ for RRA, IQR 31–119 Gy cm^2^; *P* = 0.17). Subgroup analyses revealed that the differences between left and RRA were linked to patients age (≥70 years old) and to operator experience.

The REVEREE (Randomized Evaluation of Vascular Entry Site and Radiation Exposure) trial [30] evaluated radiation exposure during cardiac catheterization comparing TFA with LRA and RRA. This study enrolled 1493 patients undergoing cardiac catheterization that were randomized in a 1:1:1 fashion. The primary endpoint was air kerma, the secondary endpoints included DAP, fluoroscopy time, operator dose per procedure, number of cineangiograms, and number of catheters. Results showed no significant differences among the three group concerning the primary endpoint (medians: TFA: 421 mGy, IQR 337–574 mGy; LRA: 454 mGy IQR: 331–643 mGy; RRA 483 mGy, IQR: 382–592 mGy; *P* = 0.146), and DAP (medians: TFA: 25.5 Gy cm^2^ IQR: 19.6–34.5 Gy cm^2^; LRA: 26.6 Gy cm^2^ IQR: 19.5–37.5 Gy cm^2^; RRA 27.7 Gy cm^2^, IQR: 21.9–34.4 Gy cm^2^; *P* = 0.40), or fluoroscopy time (medians: TFA 1.3 min IQR: 1.0–1.7 min; LRA: 1.3 min, IQR: 1.0–1.7 min, and RRA: 1.32 min, IQR: 1.0–1.7 min; *P* = 0.19). Conversely operator exposure was significantly higher in the LRA group (3 mrem, IQR: 2– 5 mrem) compared with TFA (2 mrem, IQR: 2–4 mrem; *P* = 0.001) and TRA (3 mrem, QR: 2 to 5 mrem; *P* = 0.0001).

The RAD-MATRIX [31], a sub-study of the MATRIX [10] investigated if TRA access procedures compared to TFA increases the radiation exposure risk of operator or patient. A total of 18 expert operators, performing 777 procedures in 767 patients were recruited and randomized to TRA or TFA. Each operator was equipped with lithium fluoride thermoluminescent dosimeter to measure radiation exposure on the thorax (primary endpoint), on the wrist and on the head (secondary endpoints). Results showed an equivalent dose at the thorax significantly higher with TRA (77 μSv, IQR: 39.9 μSv to 112 μSv) than TFA access (41 μSv, IQR: 23.4 mSv to 58.5 μSv; *P* = 0.019) which was still significant after normalization of operator radiation dose by fluoroscopy time or DAP. Otherwise, there was no difference in radiation dose at wrist or head among the two cohort. Similar thorax operator dose was observed for RRA (84 μSv) and LRA (52 μSv; *P* = 0.15). Operator effective dose was significantly higher with RRA than LRA (2.6 vs. 1.6 μSv; *P* = 0.016). A subsequent analysis [32] was performed to appraise the determinants of operator radiation overexposure with RRA. Operator radiation exposure was investigated in relation to the right arm position and the upper leaded glass size. Results showed a greater-than-10-fold difference in radiation at thorax level among the 14 operators who agreed to participate (from 21.5 to 267 μSv and from 0.35 to 3.5 μSv/Gy*cm^2^ after DAP normalization). Thorax dose was higher among operators who positioned the instrumented right arm far from the body (110.4 μSv, interquartile range 71.5–146.5 μSv), when compared to those that placed the instrumented right arm adjacent to the right leg (46.1 μSv, 31.3–56.8 μSv; *P* = 0.02) and the difference persisted after normalization by DAP (*P* = 0.028). In the same way, smaller full glass shield was associated with a higher radiation exposure when compared to a larger composite shield (147.5 and 60 μSv, respectively, *P* = 0.016).

Apart from the evident impact on vascular complications and access-site bleeding, the implementation of the radial access showed several additional clinical benefits [33,34]. In the AKI-MATRIX [35] study, the radial vs. femoral access impact on post-procedural acute kidney injury was explored. Renal function evaluation regarded 8210 patients, 4109 in the TRA cohort and 4101 in the TFA cohort. The primary endpoint was acute kidney injury defined as an absolute (>0.5 mg/dl) or a relative (>25%) increase in serum creatinine. Results showed an overall lower acute kidney injury rate with TRA compared to TFA (15.4% vs. 17.4%, OR 0.87; 95% CI: 0.77 to 0.98; *P* = 0.0181). In particular, a >25% relative serum creatinine increase regarded the 15.4% of the TRA cohort patients and the 17.3% of the TFA cohort patients (OR: 0.87; 95% CI: 0.77 to 0.98; *P* = 0.0195), while a >0.5 mg/dl absolute serum creatinine increase occurred in the 4.3% of patients in the TRA cohort and in the 5.4% of patients in the TFA cohort (OR: 0.77; 95% CI: 0.63 to 0.95; *P* = 0.0131). By applying the Kidney Disease Improving Global Outcomes criteria, acute kidney disease rate was 3-fold less prevalent with TRA access (OR: 0.85; 95% CI: 0.70 to 1.03; *P* = 0.090), with stage 3 acute kidney injury in the 0.68% of patients in the TRA cohort and in the 1.12% of patients in the TFA cohort (*P* = 0.0367). Post-intervention dialysis was needed in the 0.15% of patients in the TRA cohort and in the 0.34% of patients in the TFA cohort (*P* = 0.0814).

A subsequent analysis evaluated how radial and femoral access relate to bleeding, acute kidney injury and all-cause mortality to clarify the correlation between these different types of events [36]. A multistate and competing risk models highlighted large relative risk reductions in mortality for TRA compared with TFA access for the transition from acute kidney injury to death (HR 0.55, 95% CI 0.31–0.97) and for the pathway from PCI to acute kidney injury to death (HR 0.49, 95% CI 0.26–0.92). On the contrary, there was little evidence for a difference between TRA and TFA groups for the transition from bleeding to death (HR 1.05, 95% CI 0.42–2.64) and the pathway from PCI to bleeding to death (HR 0.84, 95% CI 0.28–2.49). These results suggest that radial approach benefit on mortality is mainly driven by acute kidney injury prevention while bleeding role in this association was minor [33,36].

## 4. The Distal Radial Approach

**Figure 2:**
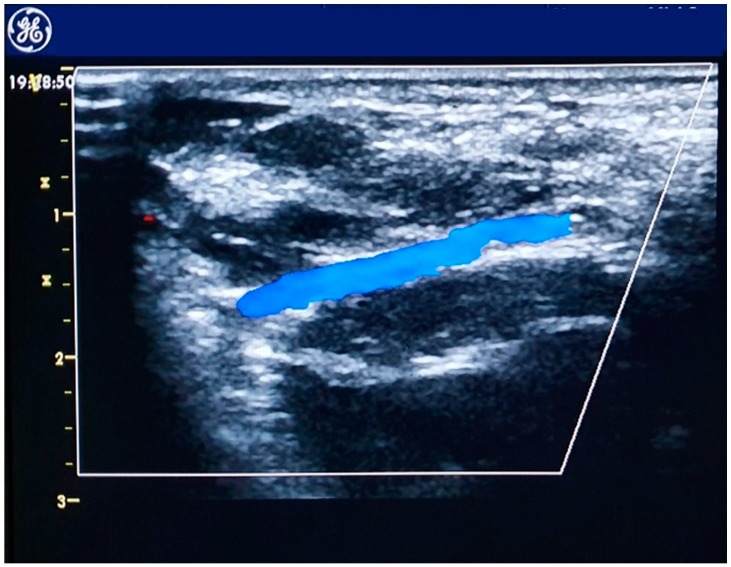
Echocolordoppler image of the distal radial artery.

The distal radial artery (Figure 2) approach was first introduced by anesthesiologists for perioperative patient monitoring [37] at the anatomical snuffbox or at the dorsum of the hand. This access is obtained where the vessel runs superficially, at the intersection of the thumb and first finger over the bony structures of the snuffbox or at the dorsum of the hand, distal to the tendon of the extensor pollicis longus muscle. The technique described by Kiemeneij [38] and Davies and Gilchrist [39] starts with a subcutaneous injection of 1–5 cc of local anesthetic, then the artery is punctured with a 21-gauge open needle, directed to point of highest pulsation, from lateral to medial with a 30–45° angle. When the blood flows through the needle a flexible, soft, J-shaped 0.21 metallic wire is inserted and the needle is removed. A small skin incision before introducer placement is sometimes necessary because the skin is thicker and harder than at the forearm [40]. A through-and-through technique should be avoided, especially if the artery is approached at the snuffbox, for the high risk of puncturing the periosteum of the scaphoid or trapezium bones, which can be painful [38]. Hemostasis is obtained compressing the artery against the base of the 1st or 2nd metacarpal bone and an elastic bandage might be necessary [39]. Theorized advantages of distal radial approach are a decreased risk of radial artery occlusion at the forearm, lower bleeding risk, lower vascular access-site complication rate [41] and an improved operator and patient comfort, especially when approaching left radial artery [40]. Ongoing randomized controlled trials are evaluating the feasibility and safety of distal radial approach compared to radial approach in various settings, including patients with STEMI [42].

## 5. Radial Artery Approach Limitations

Radial artery occlusion (RAO) after radial artery interventions remains an important issue after TRA. Despite this did not show a significant impact of hand ischemia, RAO may prevent future use of the radial artery for interventional purposes, for using the radial artery as an arterial conduit for CABG or in the need for fistula for dialysis.

For this reason, several studies evaluated strategies to reduce the risk of RAO and optimize radial puncture.

The PROPHET (Prevention of radial artery occlusion-patent hemostasis evaluation trial) study [43] enrolled 436 consecutive patients undergoing cardiac catheterization which were randomized to conventional compression or compression maintaining radial artery patency using plethysmography. Results showed that implementation of patent hemostasis is highly effective in reducing RAO (59% decrease at 24 h and 75% decrease at 30 days; *P* < 0.05).

The PROPHET-II study [44] investigated whether ipsilateral ulnar compression increases radial artery flow and could impact the incidence of RAO. This trial enrolled 3000 patients undergoing diagnostic cardiac catheterization, which were randomized to standard patent hemostasis or prophylactic ipsilateral ulnar compression in addition to patent hemostasis. Plethysmography was used to evaluate radial artery patency resulting in a lower RAO occurrence at 30 day in patients with patent hemostasis and prophylactic ulnar compression (0.9% vs. 3.0%; *P* = 0.0001).

The effect of duration of hemostatic compression on RAO was evaluated in a retrospective study [45] that involved 400 consecutive patients undergoing transradial PCI. Half of patients received hemostatic compression for 6 h after the procedure, and the other group for 2 h. Shorter duration of hemostatic compression was associated with a lower incidence of early RAO (5.5% vs.12%; *P* = 0.025) and chronic RAO (3.5% vs. 8.5%; *P* = 0.035) without differences in bleeding complications although maintaining radial patency during hemostatic compression, eliminates the adverse effect of duration of compression.

Use of anticoagulants, administered directly into the radial artery or intravenously, during the procedure has been demonstrated to reduce the incidence of RAO [46,47], particularly a dose-dependent effect of unfractionated heparin [48,49].

The artery spasm may determine friction between the artery wall and devices used during TRA and this endothelial stress can contribute to RAO development. For this reason, the use of vasodilators has been proposed. Nitroglycerin administrated into radial artery before sheath removal was associated with RAO incidence reduction [50], as well as and the use of verapamil or verapamil in combination with nitroglycerin is effective in preventing radial artery spasm [51].

On top of the added risk of long-term RAO, the radial artery has some intrinsic anatomical limitations that impedes its use in specific types of intervention. The smaller caliber of the artery, which is 2.5–3.5 times smaller than the common femoral artery, restrict its use in case of larger devices. For this reason, the femoral artery remain the default access in procedures where bulky device are needed such as transcatheter aortic valve implantation (TAVI) [52], mechanical circulatory support including intra-aortic balloon pump (IABP), Impella (Abiomed, Danvers, Massachusetts), or veno-arterial extracorporeal membrane oxygenation (ECMO) [53]. Furthermore, femoral approach is still the preferred access site for some complex procedures, particularly angioplasty of coronary chronic total occlusion [54,55].

## 6. The Ulnar Artery Approach

The ulnar artery represents an alternative access for percutaneous procedures, and it is increasingly being used when radial access is not available (Table 2) [56,57,58,59,60,61,62]. The ulnar artery stems at the inferior margin of the antecubital fossa as a large branch of the brachial artery. It passes in the forearm on the ulnar bone side deep to the flexor muscles and it reaches the hand through the Guyon canal. As compared to the radial artery, the ulnar artery has a lower incidence of anatomical variations, loops, and tortuosity; furthermore, the ulnar artery has a modest concentration of adrenergic receptors that limit the arterial spasm [60]. Conversely the deeper position of the vessel and the absence of a bony structure to compress against expose to a higher rate of access-site complication and impose a heavier compression [58]. The access technique for the ulnar artery is similar to the radial, and both the Seldinger technique and the modified Seldinger technique can be used. The ulnar nerve passes lateral to the ulnar artery, so it is recommended to start the puncture on the medial side of the ulnar artery to avoid pain and spasm.

The PCVI-CUBA [63], a single-center randomized study, compared efficacy and safety of the transulnar access (TUA) and the TRA in patients undergoing coronary angiography and PCI. This study enrolled 431 patients who were randomized to TUA (*N* = 216) or TRA (*N* = 215). Results showed no difference among the two groups for successful vascular access (93.1% in the TUA group vs. 95.5% in the TRA group; *P* = 0.84), procedural success (95.2% in the TUA group vs. 96.2% in the TRA group; *P* = 0.82), arterial occlusion (5.7% in the TUA group vs. 4.7% in the TRA group, *P* = 0.76) and major bleeding (5.5% in the TUA group vs. 8.1 in the TRA group, *P* = 0.47). Procedural time (mean 14 ± 8.2 min vs. 12.7 ± 6.7 min *P* = 0.06), fluoroscopy time (mean 5.6 ± 5.1 min vs. 5.2 ± 4.2 min, *P* = 0.35) and X-ray DAP (mean 7559 ± 4865 mGy/cm^2^ vs. 7195 ± 4850 mGy/cm^2^ *P* = 0.43) were similar between the two group. Freedom from MACE at 30 days was comparable between the two groups (97.8% for ulnar group and 95.8% for radial group, *P* = 0.41).

Another single-center, randomized study [64] evaluated the safety and efficacy of the TUA versus the TRA for coronary angiography and PCI. This study randomized 240 patients undergoing coronary angiography, followed or not by intervention. Results showed a comparable artery stenosis rate between the two groups at 1 day (11.0% in TUA group vs. 12.3% in the TRA group; *P* = 0.758) and at 30 days (5.1% in the ulnar group vs. 6.6% in the radial group; *P* = 0.627). Arterial occlusion rate was similar in the two cohort at 1 day (5.1% in the TUA group vs. 1.7% in the TRA group, *P* = 0.627) and at 30 days (6.6% in the TUA group vs. 4.9% in the TRA group; *P* = 0.164). Minor bleeding was similar between the two groups (5.9% in the TUA group vs. 5.7% in the TRA group; *P* = 0.949).

In contrast to these single-center studies, the AURA of ARTEMIS trial [62] failed to demonstrate the non-inferiority of a default TUA compared to TRA. This prospective randomized multicenter study enrolled 902 patients undergoing diagnostic coronary angiography and PCI who were randomized to TRA (*N* = 440) or TUA (*N* = 462). The primary endpoint was a composite of crossover to another arterial access, MACE, and major vascular complications of the arm at 60 days. The trial was early stopped because the first interim analysis showed the inferiority of the TUA for primary outcome (42.2% vs. 18.0%; *P* < 0.0001). After adjustment for operator clustering, the difference in the primary endpoint became inconclusive (24.30%; 99.99% CI, −7.98% to 56.58%; *P* = 0.03 at α = 0.0001). Moreover, the crossover rate, which driven most of the difference for the primary endpoint, was higher in the TUA group, with a difference of 26.34% (95% CI, 21.53%–31.27%; *P* < 0.001).

A subsequent single-center randomized study [59] including 535 consecutive patients evaluated safety and feasibility of TUA for coronary catheterization. The primary endpoints were the success of artery cannulation and the access-site-related complications at day 30 (hematoma, artery stenosis, artery occlusion, arteriovenous fistula, pseudoaneurysm, and nerve injury). Results showed a comparable successful puncture rate between the two cohort (91.5% in the TUA group vs. 95.1% in TRA group; *P* > 0.05). Hematoma occurrence was similar in between the two groups (7.7% in the TUA group vs. 4.2% in the TRA group; *P* = 0.100). Ulnar nerve injury occurred in one patient in the TUA group. Asymptomatic arterial occlusion rate was similar in the two group (1.1% in the TUA group vs. 3.0% in TRA group; *P* = 0.137). Freedom from MACE rate was also similar between the two group (90.6% in the TUA vs. 91.3% in the TRA group; *P* = 0.985).

A randomized single-center, trial [65] compared the clinical outcomes of 445 patients undergoing coronary artery intervention with TUA (*N* = 220) and TRA (*N* = 225). At 1-year follow-up, the primary outcome, arterial occlusion of a forearm artery, was lower in the TUA group (2.7% vs. 9.3%; *P* = 0.007). The occlusion rate of approached vessel was lower in TUA group (OR 3.85, *P* 0.006). A higher of incidence of hematomas (13.2% vs. 5.8%, *P* = 0.01) and symptoms of discomfort (15.5% vs. 5.8%, *P*= 0.002) emerged in the TUA group.

Finally, the AJmer ULnar ARtery Working Group Study (AJULAR) study [56], a single-center, randomized study, tested the non-inferiority on TUA as compared to TRA when performed by an experienced operator (at least 150 TRA/year and 50 TUA performed). This study enrolled 2532 patients undergoing cardiac catheterization who were randomized to TUA (*N* = 1270) or TRA (*N* = 1262). The primary endpoint was a composite of MACE during hospital stay, crossover to another arterial access, major vascular events during hospital stay (large hematoma with hemoglobin drop of ≥3 g%) or vessel occlusion rate. Secondary endpoints were each component of the primary endpoint, failed attempts (≥3 attempts prior to successful cannulation), procedural and fluoroscopy times, contrast volume used, and vasospasm. Results showed a similar occurrence of primary endpoint (14.6% in TUA group vs. 14.4% in TRA group; RR: 1.01; 95% CI: 0.83–1.2; *P* = 0.92 at α = 0.05). The secondary endpoints were all comparable among the two groups, except large hematoma, for which non-inferiority could not be proved. Twelve patients in the TUA group suffered transient paresthesia.

A meta-analysis [66] of six randomized controlled of TUA vs. TRA has been presented, including 5299 patients undergoing percutaneous coronary procedure. Results showed a comparable incidence of MACE between the two access sites (RR 0.90; 95% CI 0.66–1.23) but an excess of vascular complications in the TUA group (RR 3.58; CI 2.67–4.79, *P* < 0.00001). No difference for arterial access time, fluoroscopy time, and contrast load was observed.

## 7. Radial Access for Non-Coronary Cardiovascular Interventions

The exponential growth of the technique expanded TRA use also to endovascular peripheral artery disease interventions and some selected structural heart disease interventions [66,67,68] TRA has been reported as primary access-site for alcohol septal ablation [69] paravalvular leak closure [70], ventricular septal defect closure [71], or balloon aortic valvuloplasty (BAV) [72]. Also, more extensively, TRA has been used as secondary access for transcatheter aortic valve replacement (TAVR), patent ductus arteriosus closure, and endovascular repair of abdominal or thoracic aneurysm [68].

In the domain of structural heart interventions, BAV can be a useful tool in patients with severe aortic stenosis who require urgent non-cardiac surgery or safely bridge to either to Surgical Aortic Valve Replecement (SAVR) or TAVR [72]. In addition, population’s mean age in western countries is increasing and it is increasingly important to avoid futile TAVR procedures. Still, there is no clear evidence on which patients should be preliminarily excluded from aortic valve intervention because of excessive risk or clinical futility. In this setting BAV as bridge to TAVR, might serve as a stratification method in uncertain TAVR recipients.

Yet, considering the higher rate of comorbidities in this frail population, the rate of vascular complications and the relative attributable mortality remain high [73].

For this reason, using alternative vascular approaches, which may reduce the risk of such complications is attractive. Mini-invasive radial BAV reduces the rate of vascular complications with feasibility and efficacy comparable with standard femoral approach [74].

Implementation of large-bore access sites from the femoral artery and increasing sheath-artery diameter ratio invariably increases the rate of vascular complications [75]. Moreover, during transfemoral TAVR, CT studies of the ilio-femoral arteries confirmed the need for a <1:1 lumen to sheath ratio to avoid risk of vascular damage and serious adverse events [72].

Yet, owing to specific histologic characteristics of the radial artery wall, the 1:1 safety rule could be less important in this scenario. The radial artery wall is thicker than that of other arteries [76] and the prevalent muscular tunica has unique elastic features resulting more compliant than the other arterial vessels, in which progressive elastic fiber degeneration often increases stiffness in older patients [77].

These features, together with the more superficial position and the easier compression, could explain the lower incidence of vascular complications during radial BAV.

The first step to perform radial BAV is obtaining two radial accesses with standard technique, or ulnar access in case radial access would be unavailable. In case only one radial access is possible a mono-lateral approach could be performed, even if two different arterial accesses provide opportunity to switch quickly to the contralateral access, check the hemodynamic efficacy of rapid pacing and the possibility to inject contrast.

**Figure 3:**
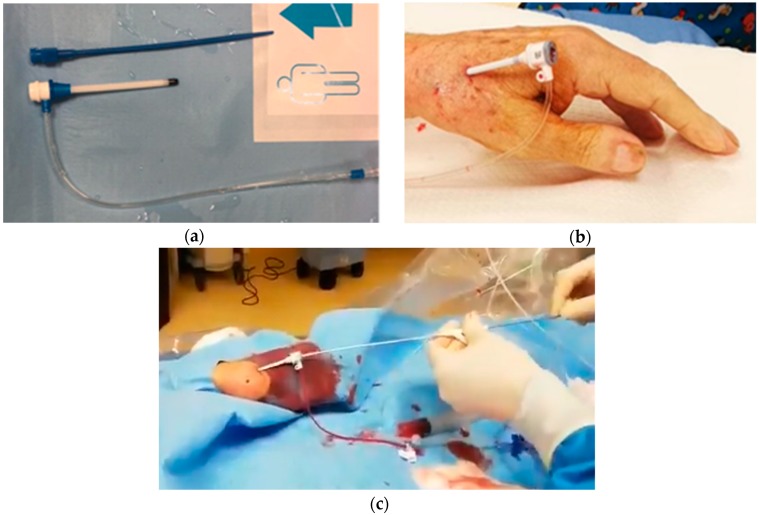
Vascular access for radial balloon aortic valvuloplasty. (**a**) Brite Tip 5.5 cm pediatric 8 French sheath (CORDIS, Ireland). (**b**) regular 11 cm 8F sheath, 5 cm out of the distal radial artery. (**c**) Vascular access for radial balloon aortic valvuloplasty: “Push and pull” maneuver for balloon retrieval.

After obtaining vascular accesses, the aortic valve should be crossed with conventional 0.035″ guidewire. Then a pigtail catheter is advanced in the left ventricle and basal peak-to-peak and mean gradients are measured. In this phase, vascular tortuosity of the epi-aortic branches could hinge on catheter control. In this case, switching from one radial access to the contralateral could be useful. After placing a stiff 0.035″ guidewire in the left ventricle carefully avoiding mitral chordae along the path, the 6 French sheath should be exchanged with an 8 or 9 French femoral sheath (depending on the compatibility of the aortic balloon’s manufacturer used). A very short sheath is preferable (5.5 cm) but longer sheaths can be equally used if they are advanced no more than 5–6 cm inside the artery (Figure 3a–b).

When the sheath is in place, advancement of the aortic valvuloplasty balloon is usually very easy. When the balloon is across the aortic valve the two electric alligator-connectors should be connected to the distal end of the stiff guidewire placed inside the left ventricle and to a needle placed on the patient’s skin and connected to a temporary pacemaker [78]. Alternatively, a transvenous temporary pacemaker stimulating in the right ventricle could be placed through the standard routes. One rapid 160–200 bpm burst should be attempted before the balloon is advanced through the valve to test pacing efficacy. Once the balloon is advanced through the aortic valve and rapid pacing is started the balloon is inflated making sure to maintain position inside the valve. When several inflations have been performed (usually 2–3), the balloon should be exchanged with a pigtail catheter to measure the final pressure gradient.

The balloon could be then retrieved: this is usually easy until it reaches the radial artery. Ideally, two operators should coordinate a “push and pull” maneuverer to minimize the friction of the balloon on the arterial wall in this phase (Figure 3c) and perform a gentle retrieval through the sheath. At this stage of the procedure the patient may complain of acute but transient pain in the forearm region.

It is advisable to perform a careful hemostasis of the radial artery to minimize the risk of occlusion. Good results have been described with patent hemostasis.

According to the experience gained with the radial BAV pilot study and the subsequent ongoing multicenter SOFTLY2 Register, mini-invasive radial BAV is feasible in most patients, regardless of the body weight. Nevertheless, there are some contraindications to the procedure that, even if uncommon, they should to be highlighted: first, the presence of a very small artery with a high take-off of the artery above the elbow or a diffusely diseased radial artery, which is often discovered after several failed attempt to advance diagnostic catheters over the elbow. In addition, severe loops or accessory radial artery anatomy, could increase the risk of arterial rupture. Finally, the evidence of Monkeberg’s disease of the forearm arteries (extended calcifications) should discourage any procedure’s attempt.

## 8. Conclusions

TRA is now established as the standard of care access for percutaneous coronary diagnostic and intervention. While TRA already demonstrated a sizable benefit in terms of hard endpoints compared to TFA, a continuously growing body of evidence is focused on surpassing current TRA limitations and expanding alternative vascular accesses, as TUA or distal radial artery for cardiovascular interventions. The recent implementation of TRA as primary or secondary access site for peripheral and structural interventions might provide some of the advantages already observed in the coronary domain also in these new techniques. Nevertheless, owing to the intrinsic anatomical limitations of the TRA preventing large-bore device use, mastering best practices for femoral access remain a priority for future generations of interventional cardiologists.

## Figures and Tables

**Table 1:** Clinical studies for Radial vs. Femoral approach in percutaneous coronary intervention.

Trial, Year	N. of Patients	STEMI%	NSTE-ACS%	All-Cause Mortality%	MACE%	Access-Site Bleeding%	Major Bleeding%
RIVAL, 2011							
Radial	3507	27	73	1.3	3.2	1.4	0.7
Femoral	3514	29	71	1.5	3.2	3.7	0.9
RIFLE-STEACS, 2012							
Radial	500	100	-	5.2	7.2	2.6	1.8
Femoral	501	100		9.2	11.4	6.8	2.8
STEMI-RADIAL, 2014							
Radial	348	100	-	2.3	3.4	0.6	1.4
Femoral	359	100		3.1	4.2	5.3	7.2
MATRIX, 2015							
Radial	4197	48	52	1.6	8.8	0.4	1.5
Femoral	207	48	52	2.2	10.2	1	2.3
SAFARI-STEMI, 2019							
Radial	1136	100	-	1.5	4	-	1.1
Femoral	1156	100		1.3	3.4		1.3

STEMI: ST-Elevation myocardial infraction. NSTE-ACS: non-ST-Elevation acute coronary syndrome. MACE: major adverse cardiovascular events.

**Table 2:** Clinical studies of transradial vs. transulnar approach.

First Author, Year	N. of Patients (TUA/TRA)	STEMI% (TUA/TRA)	NSTEMI% (TUA/TRA)	Successful Vascular Access% (TUA/TRA)	Crossover to Other Approach% (TUA/TRA)	Arterial Access Time (min) (TUA/TRA)	PCI% (TUA/TRA)	Bleeding or Hematoma% (TUA/TRA)	Occlusion% (TUA/TRA)	MACE% (TUA/TRA)
Aptecar, 2006	216/215	17.5/16.7	22.5/27.6	93.1/95.5	6.9/4.2	NR	43.5/44.2	6.8/8.1	5.7/4.7	2.1/4.2
Li, 2010	118/122	4.2/4.9	77.1/73	98.3/100	1.7/0	NR	65/69.2	5.9/5.7	1.7/4.9	0/0
Hahalis, 2013	462/440	14.1/13.2	37.4/38.4	67.7/99.1	32.3/5.9	NR	36.4/32.7	3.2/0.5	10.4/4.3	2.8/3.4
Geng, 2014	271/264	14.4/13.6	72.7/70.4	91.5/95.1	8.5/4.2	NR	62.4/52.3	13.2/7.9	1.9/5.8	9.4/8.7
Liu, 2014	317/319	19.6/20.4	75.9/77.4	92.7/95.9	1.6/1.9	6.3 ± 1.3/5.9 ± 1.2	100/100	4.1/9.4	6.3/4.7	1.9/2.5
Gokhroo, 2016	1270/1262	48.3/45.2	27.6/30.6	95.6/96.2	4.4/3.8	5.6 ± 2.1/5.9 ± 1.7	NR	6.2/6.7	6.1/6.6	2.9/3.2
Bi, 2017	220/225	NR	NR	90.9/92.9	9.1/7.1	NR	66.8/62.2	28.6/11.5	2.7/9.3	NR

STEMI: ST-Elevation myocardial infarction. NSTEMI: non-ST-Elevation myocardial infarction. MACE: major adverse cardiovascular events.
